# Association between triglyceride–glucose index trajectories and radiofrequency ablation outcomes in patients with stage 3D atrial fibrillation

**DOI:** 10.1186/s12933-024-02219-w

**Published:** 2024-04-05

**Authors:** Sixiang Jia, Yanping Yin, Xuanting Mou, Jing Zheng, Zhe Li, Tianli Hu, Jianqiang Zhao, Jiangbo Lin, Jiaqi Song, Fanli Cheng, Yiran Wang, Kaini Li, Wenting Lin, Chao Feng, Weili Ge, Shudong Xia

**Affiliations:** 1 Department of Cardiology, International School of Medicine, the Fourth Affiliated Hospital of School of Medicine, International Institutes of Medicine, Zhejiang University, Yiwu, 322000 China; 2 Department of Cardiology, Taizhou Hospital of Zhejiang Province affiliated to Wenzhou Medical University, Dongdu Road Linhai, Linhai, Zhejiang Province 317000 China; 3 Laboratory of Cardiovascular Disease, Taizhou Hospital of Zhejiang Province affiliated to Wenzhou Medical University, Linhai, Zhejiang Province 317000 China; 4 QuzhouPeoplès Hospital, The Quzhou Affiliated Hospital of Wenzhou Medical University, Quzhou, Zhejiang Province 324000 China; 5 Department of Endocrinology, Yiwu Central Hospital, Yiwu, 322000 China

**Keywords:** Atrial fibrillation recurrence, Triglyceride–glucose index trajectory, Insulin resistance, Radiofrequency catheter ablation

## Abstract

**Background:**

This study investigates the relationship between triglyceride-glucose (TyG) index trajectories and the results of ablation in patients with stage 3D atrial fibrillation (AF).

**Methods:**

A retrospective cohort study was carried out on patients who underwent AF Radiofrequency Catheter Ablation (RFCA) at the Cardiology Department of the Fourth Affiliated Hospital of Zhejiang University and Taizhou Hospital of Zhejiang Province from January 2016 to December 2022. The main clinical endpoint was determined as the occurrence of atrial arrhythmia for at least 30 s following a 3-month period after ablation. Using a latent class trajectory model, different trajectory groups were identified based on TyG levels. The relationship between TyG trajectory and the outcome of AF recurrence in patients was assessed through Kaplan-Meier survival curve analysis and multivariable Cox proportional hazards regression model.

**Results:**

The study included 997 participants, with an average age of 63.21 ± 9.84 years, of whom 630 were males (63.19%). The mean follow-up period for the participants was 30.43 ± 17.75 months, during which 200 individuals experienced AF recurrence. Utilizing the minimum Bayesian Information Criterion (BIC) and the maximum Entropy principle, TyG levels post-AF RFCA were divided into three groups: Locus 1 low-low group (*n* = 791), Locus 2 low-high-low group (*n* = 14), and Locus 3 high-high group (*n* = 192). Significant differences in survival rates among the different trajectories were observed through the Kaplan-Meier curve (*P* < 0.001). Multivariate Cox regression analysis showed a significant association between baseline TyG level and AF recurrence outcomes (HR = 1.255, 95% CI: 1.087–1.448). Patients with TyG levels above 9.37 had a higher risk of adverse outcomes compared to those with levels below 8.67 (HR = 2.056, 95% CI: 1.335–3.166). Furthermore, individuals in Locus 3 had a higher incidence of outcomes compared to those in Locus 1 (HR = 1.580, 95% CI: 1.146-2).

**Conclusion:**

The TyG trajectories in patients with stage 3D AF are significantly linked to the outcomes of AF recurrence. Continuous monitoring of TyG levels during follow-up may help in identifying patients at high risk of AF recurrence, enabling the early application of effective interventions.

**Supplementary Information:**

The online version contains supplementary material available at 10.1186/s12933-024-02219-w.

## Introduction

Atrial fibrillation (AF) is a prevalent tachyarrhythmia characterized by rapid and disordered atrial electrical activity [1–3], substantially increasing the risk of mortality, stroke, and heart failure [1, 4–7]. The global incidence and prevalence of AF continue to increase, with projections indicating that by 2050, Asia alone may host more than 72 million AF patients [8, 9].

Studies such as CAPTAF, EAST-AFNET 4, and CABANA have provided substantial evidence in favor of radiofrequency catheter ablation (RFCA) as a highly effective treatment for atrial fibrillation (AF), outperforming antiarrhythmic drugs in effectively managing rhythm and slowing disease progression, thereby securing its position in clinical practice [10–12]. Despite this success, post-RFCA challenges persist, primarily due to a significant recurrence rate; statistics indicate that within one year post-treatment, approximately 30–50% of patients experience AF relapse [1, 13–15].

In recent years, various scoring systems have been developed to assess the risk of AF recurrence, aiming to address its high relapse rate. These tools, including APPLE, CAAP-AF, CHA2DS2-VASc, HATCH, and DR-FLASH, effectively identify factors associated with recurrent AF [16–20]. While studies have suggested biomarkers like BNP and homocysteine as reliable predictors for AF ablation outcomes, there remains no consensus among electrophysiologists on their integration with clinical scoring systems [15, 21, 22].

The Triglyceride-Glucose (TyG) index, a notable biochemical marker assessing insulin resistance (IR), has drawn attention for its association with increased cardiovascular disease risks such as stroke, heart failure, and myocardial infarction [23–27]. Significant evidence has linked higher rates of AF occurrence to IR, with animal studies illustrating how IR affects atrial structure and function, intracellular calcium homeostasis, and glucose transporter expression, thereby increasing susceptibility to AF [28, 29]. Furthermore, IR is known to contribute to oxidative stress, inflammation, neurohumoral activation, and interstitial fibrosis, all of which increase the vulnerability of the atrial substrate to AF development [2, 30, 31]. Tang et al. identified that preoperative TyG levels were associated with AF recurrence in non-diabetic RFCA patients, highlighting the impact of IR-induced changes on atrial structure and electrophysiology due to abnormal glucose metabolism [32]. Previous research primarily focused on single baseline TyG measurements, potentially overlooking the sustained impact of TyG exposure on post-AF ablation prognosis. The exploration of dynamic TyG trajectory post-RFCA in AF patients represents a new area of research.

The most recent 2023 AHA guidelines introduced a novel phase classification for AF, categorizing patients into four stages: “At risk for AF,” “Pre-AF,” “AF,” and “Permanent AF.” Patients who have undergone successful percutaneous or surgical intervention to eliminate AF are classified into the 3D stage [1]. The blanking period is defined as the three-month period following AF ablation. This study aimed to investigate the significance of TyG index trajectory during the blanking period following AF RFCA.

## Methods

### Study design and populations

The study cohort was gathered from two Class-A tertiary healthcare facilities in China—the Fourth Affiliated Hospital of Zhejiang University School of Medicine and Taizhou Hospital in Zhejiang Province. This retrospective investigation included 1372 individuals diagnosed with atrial fibrillation (AF) who received radiofrequency catheter ablation (RFCA) treatment from January 2016 to December 2022. Participants’ ages varied between 23 and 89 years. It is important to note that all patients were treated by the institution’s skilled clinical electrophysiology team.

**Fig. 1:**
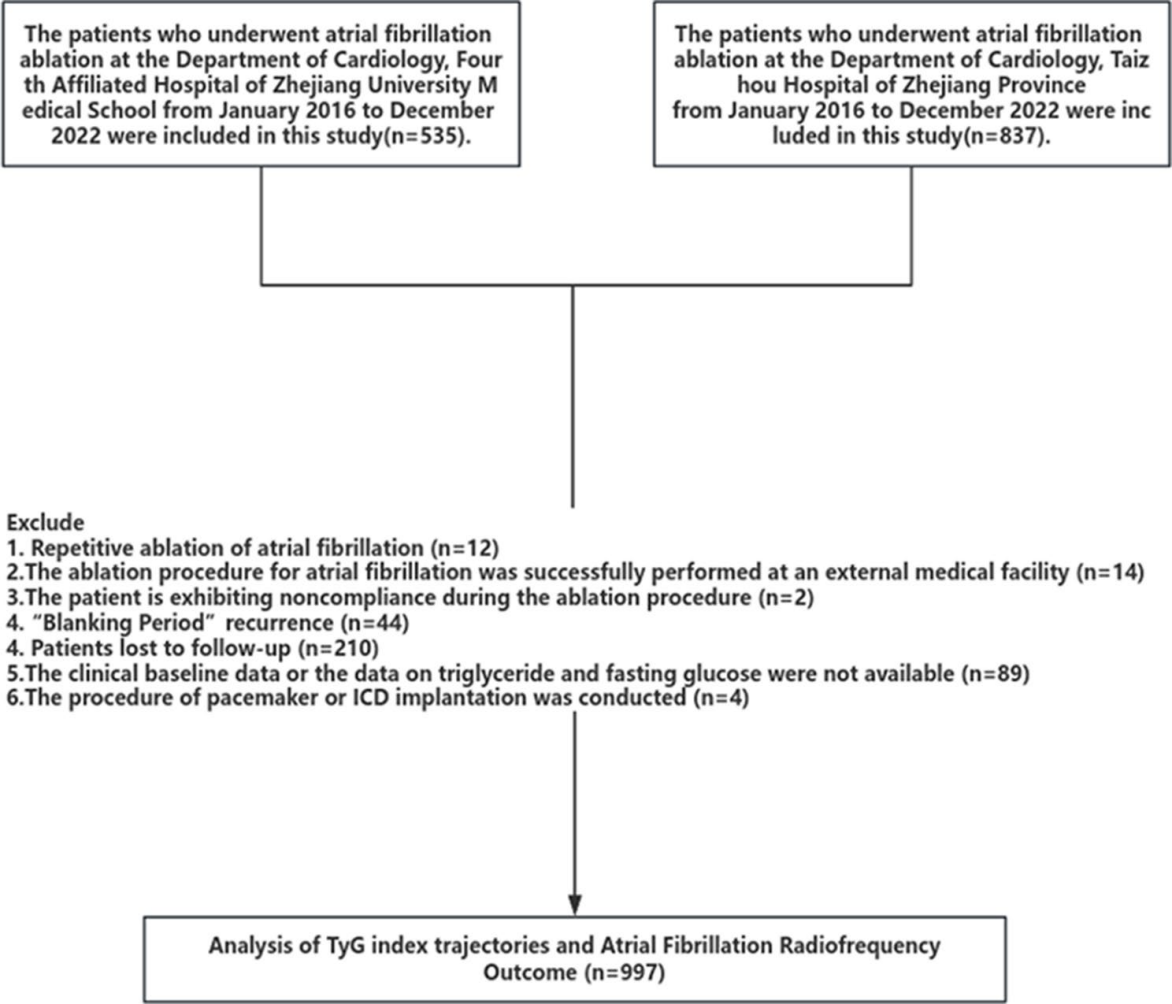
Study flow chart

Then, we excluded participants who met the following criteria:

1. Patients lost to follow-up
2. patients with fewer than 3 medical records or deficient serum glucose and triglyceride levels upon admission
3. Patients with diseases such as acute myocardial infarction, valvular heart disease, malignant tumors, hyperthyroidism, etc
4. The reoccurrence time of AF was less than 3 months (“Blanking Period”)
  The recurrence of AF 3 months post-ablation served as the primary outcome, and the follow-up deadline was December 2023. After the exclusion criteria were met, a cohort of 997 AF patients was included in the study. The research received approval from both the Ethics Committee of the Fourth Affiliated Hospital of Zhejiang University School of Medicine (No.2024047) and Taizhou Hospital of Zhejiang Province (No.KL20240156), which waived the requirement for patient informed consent. Figure 1 shows the flowchart of this study

### Data collection and definition

The blank period was defined as the post-RFCA time interval, typically lasting for 3 months.

The endpoint event is identified as AF recurrence following RFCA, with AF recurrence characterized by the presence of sustained atrial tachyarrhythmias (atrial fibrillation or flutter, atrial tachycardia) enduring for 30 s or more, as documented by 12-lead electrocardiography or 24-h Holter monitoring during the blank period.

Height and weight measurements were conducted using an automated device while participants stood upright without wearing shoes. The Body Mass Index (BMI) was determined by dividing weight (kg)/height (m^2^). Hemodynamic variables, namely Systolic Blood Pressure (SBP) and Diastolic Blood Pressure (DBP), were measured on the right arm using a sphygmomanometer after a resting period of at least 5 min.

Fasting blood samples were acquired in the morning following admission, with a minimum fasting duration of 8 h. Biochemical parameters, including fasting blood glucose (FBG), triglyceride (TG), high-density lipoprotein (HDL), albumin, creatinine (Cre), estimated glomerular filtration rate (eGFR) and NT-proBNP, were determined by standardized laboratory techniques. The concentrations of FBG and TG were collected from patients at two specific intervals: the 1st to 2nd months and the 2nd to 3rd months following radiofrequency ablation. Furthermore, the TyG index at each visit was calculated as ln (fasting TG [mg/dL] × FPG [mg/dL]/2). The left ventricular ejection fraction (LVEF) and left atrial diameter (LAD) were quantified through the application of transthoracic echocardiography.

Diabetes was defined as meeting ≥ 1 of the following criteria: a fasting glucose level ≥ 126 mg/dL (≥ 7.0 mmol/L), HbA1c ≥ 6.5%, or a self-reported physician diagnosis of diabetes, or use of antidiabetic medications. Hypertension was defined as having an SBP ≥ 140 mmHg, or DBP ≥ 90 mmHg, or documented use of antihypertensive medications, as indicated by clinical records or self-reported information. Coronary Artery Disease (CAD) was identified by the presence of stenosis exceeding 50% in the left or right main coronary artery or its major branches, confirmed through coronary angiography in prior hospitalization records. The term “incorporation of additional arrhythmias” refers to evidence of ventricular or supraventricular arrhythmias, excluding atrial fibrillation (AF). Types of AF include paroxysmal and persistent AF: paroxysmal AF is intermittent and terminates within seven days of onset, while persistent AF persists for seven days or longer and requires intervention. Furthermore, both CHA2DS2-VASc score and HAS-BLED score were calculated following guideline standards. The study documented three distinct time points: the moment of initial AF onset (T1), the time of initial acceptance of RFCA (T2), and the time of AF reoccurrence post-ablation (T3). We needed to calculate the time intervals between T1, T2, and T3, with T1-T2 defined as the onset-to-ablation time and T2-T3 defined as the recurrence time. The measurement units for these time intervals are standardized to months.

The CAAP-AF total score spans from 0 to 13 points, with the following specific scoring criteria: CAD contributes 1 point; left atrial diameter receives 0 points for < 4.0 cm, 1 point for 4.0–4.4 cm, 2 points for 4.5–4.9 cm, 3 points for 5.0–5.4 cm, and 4 points for ≥ 5.5 cm; age contributes 0 points for < 50 years, 1 point for 50–59 years, 2 points for 60–69 years, and 3 points for ≥ 70 years; persistent or long-standing persistent atrial fibrillation adds 2 points; the number of failed antiarrhythmic drugs results in 0 points for 0 types, 1 point for 1–2 types, and 2 points for > 2 types; female gender contributes 1 point.

The APPLE total score ranges from 0 to 5 points, with the following specific criteria: Age over 65 years contributes 1 point; persistent atrial fibrillation adds 1 point; impaired estimated glomerular filtration rate (eGFR) [< 60 mL/(min. 1.73 m^2^)], adds 1 point; left atrial diameter ≥ 4.3 cm contributes 1 point; left ventricular ejection fraction (LVEF) < 50% adds 1 point.

### Atrial fibrillation radiofrequency catheter ablation

The RFCA procedure was conducted under sedation, with continuous monitoring of ECG and oxygen saturation throughout the ablation process. Transesophageal cardiac ultrasound was carried out 24 h before RFCA to rule out the presence of left atrial thrombus. Standardized circumflex pulmonary vein isolation was performed on all patients. Intraoperatively, a sheath was inserted through the femoral vein, and after accessing the left atrium via septal puncture, the Carto 3 three-dimensional anatomical mapping system was utilized for precise localization during circumflex pulmonary vein isolation. Ablation power ranged from 35 to 45 W for the anterior wall and 30–40 W for the posterior wall. Additional ablation pathways, such as superior vena cava isolation, roof line or mitral isthmus line ablation of the left atrium, and tricuspid isthmus ablation, were determined by the operator based on individual requirements. Pulmonary venous potentials were recorded using a LASSO® NAV catheter before, during, and after the ablation procedure to assess its effectiveness. The ablation endpoint was achieved when the bi-directional transmission (afferent and efferent) of pulmonary venous potentials ceased completely for at least 30 min. In cases of persistent atrial fibrillation or other atrial tachycardia post-ablation, direct current cardioversion could be considered as an option to restore sinus rhythm. According to the latest guidelines, patients with successful AF RFCA have been defined as stage 3D AF.

### Management of atrial fibrillation radiofrequency ablation

Following ablation, all patients undergo outpatient follow-up and 24-hour ambulatory electrocardiogram monitoring at 1, 3, and 6 months post-procedure. At 12 months post-procedure, outpatient follow-up and 7-day long-term ambulatory electrocardiogram monitoring are conducted. Subsequently, outpatient follow-up and 24-hour ambulatory electrocardiogram monitoring are performed every 6 months.

Sequential oral anticoagulant therapy should be initiated the day after RFCA to minimize the interruption of anticoagulation treatment. According to individual CHA2DS2-VASc scores, AF patients are advised to continue oral anticoagulants for a minimum of two months [1]. Persistent AF patients are prescribed amiodarone for three months post-RFCA to uphold sinus rhythm and undergo regular monitoring for medication side effects. If there is no recurrence during the three-month blanking period, patients with paroxysmal AF should refrain from using class I or III antiarrhythmic drugs. In the event of AF recurrence, β-receptor blockers are prioritized as the first-line rate control treatment, with propafenone being considered if symptoms persist despite β-receptor blocker usage. All class I or III antiarrhythmic drugs are ceased at the conclusion of the blanking period. It is emphasized that fasting blood glucose and triglyceride levels should be measured from patients’ blood samples during the first month and either the second or third month of follow-up after ablation.

### Statistical methodology

Missing data were managed using the Random Forest algorithm with default settings, implemented in the R package “missForest.” This method, a non-parametric approach, utilizes random forest to impute missing values, making it suitable for both continuous and categorical variables. Its core algorithm involves using known variables as independent variables and constructing random forests to predict missing values using the variables containing those missing values as dependent variables [33]. Trajectory fitting was performed using the R package “LCMM,” while the optimal number of trajectory categories was determined based on the minimum Bayesian Information Criterion (BIC) and the principle of maximum Entropy. The minimum BIC helps select an optimal model from a finite set by incorporating a penalty term to counterbalance overfitting and ensure a balanced model selection approach. The principle of maximum entropy suggests choosing the model with the highest entropy when acquiring knowledge about probabilistic models among feasible distributions, within defined constraints [34–40].

TyG index was stratified according to previous literature reports from the arrhythmia center at Fuwai Hospital. The TyG index has been classified into three groups: Group 1 (TyG index < 8.67), Group 2 (TyG index between 8.67 and 9.37), and Group 3 (TyG index > 9.37) [32].

After grouping by AF RFCA outcome, the distribution of normally distributed measurement data was described using the mean ± standard deviation (SD). A t-test was utilized to compare differences between two groups of normally distributed quantitative data. M (Q1, Q3) was used to determine the distribution of nonnormally distributed measurement data, and the Wilcoxon rank sum test was applied to compare differences between two groups of skewed quantitative data. The distribution of measurement data is represented as n (%), and the chi-square test was used to compare differences between groups.

The survival distribution among AF patients across different TyG trajectories was visualized using a Kaplan-Meier survival curve, focusing on recurrence as the primary endpoint. Variations in the curves were evaluated using the Log-rank test. Potential influencing factors were identified through the univariable Cox proportional hazards model, followed by an investigation of the trajectory’s impact on outcomes using a multivariable Cox proportional hazards model. Model 1 acted as a baseline model with no covariate adjustments, while Model 2 included adjustments for Gender, Age, and BMI. Expanding on Model 2, Model 3 integrated additional factors like AF Duration, Type of AF, left atrial diameter, LVEF status, and NT-proBNP. Subgroup analyses were conducted for Age, Gender, Type of AF, Diabetes, and LVEF status.

To assess the predictive value of the TyG and TyG trajectories for AF recurrence, we compared them with conventional risk factors for AF recurrence and relapse system scores and generated a receiver operating characteristic (ROC) curve and the area under curve (AUC) values were calculated. All the statistical analyses were conducted using R software version 4.3.0, with statistical significance set at *P* < 0.05.

## Results

### Baseline data of the study cohort population

The missing data in the baseline dataset were preprocessed using missForest (refer to the Supplement). A total of 997 individuals participated in this study, with 200 showing a recurrence of AF. The average age of the participants in the study cohort was 63.21 ± 9.84 years; 630 males (63.19%) and 367 females (36.81%) were included. Grouping based on the presence or absence of recurrent AF revealed differences across variables such as AF duration, type of AF, left atrial diameter, left ventricular ejection fraction (LVEF) status, NT-proBNP levels, HDL levels, baseline TyG index, the use of Class II antiarrhythmic drugs, APPLE score, and CAAP-AF score; all these details are provided in Table 1.

**Table 1:** Baseline atrial fibrillation populations

Variable	Total (*n* = 997)	Status		Statistic	*P*
		Non recurrence (*n* = 797)	Recurrence (*n* = 200)		
Age, Mean ± SD	63.21 ± 9.84	63.17 ± 9.86	63.37 ± 9.81	t=-0.246	0.805
Gender, n (%)				χ²=1.562	0.211
Female	367 (36.81)	301 (37.77)	66 (33.00)		
Male	630 (63.19)	496 (62.23)	134 (67.00)		
BMI, Mean ± SD	24.77 ± 3.12	24.74 ± 3.09	24.86 ± 3.23	t=-0.478	0.633
AF Duration (Months), M (Q_1_;, Q_3_)	12.00 (1.00, 36.00)	12.00 (1.00, 36.00)	24.00 (2.75, 60.00)	Z=-3.725	**< 0.001**
Combined with other arrhythmias, n (%)				χ²=0.549	0.459
No	830 (83.25)	667 (83.69)	163 (81.50)		
Yes	167 (16.75)	130 (16.31)	37 (18.50)		
Hypertension, n (%)				χ²=0.271	0.602
No	485 (48.65)	391 (49.06)	94 (47.00)		
Yes	512 (51.35)	406 (50.94)	106 (53.00)		
SBP, M (Q_1_;, Q_3_)	128.00 (115.00, 140.00)	128.00 (116.00, 141.00)	125.50 (114.00, 140.00)	Z=-1.231	0.218
DBP, M (Q_1_;, Q_3_)	79.00 (71.00, 86.00)	78.00 (71.00, 86.00)	80.00 (70.75, 87.00)	Z=-0.695	0.487
Coronary Artery Disease, n (%)				χ²=0.094	0.759
No	864 (86.66)	692 (86.83)	172 (86.00)		
Yes	133 (13.34)	105 (13.17)	28 (14.00)		
Diabetes, n (%)				χ²=0.012	0.912
No	860 (86.26)	687 (86.20)	173 (86.50)		
Yes	137 (13.74)	110 (13.80)	27 (13.50)		
Type of AF				χ²=27.69	**< 0.001**
Paroxysmal	568 (56.97)	487 (61.10)	81 (40.50)		
Persistent	429 (43.03)	310 (38.90)	119 (59.50)		
CHA2DS2-VASc, M (Q_1_;, Q_3_)	2.00 (1.00, 3.00)	2.00 (1.00, 3.00)	2.00 (1.00, 3.00)	Z=-0.651	0.515
HAS-BLED, M (Q_1_;, Q_3_)	1.00 (0.00, 1.00)	1.00 (0.00, 1.00)	1.00 (0.00, 1.00)	Z=-0.733	0.464
Left atrial diameter, M (Q_1_;, Q_3_)	39.00 (34.00, 43.00)	38.00 (34.00, 42.00)	42.00 (37.00, 47.00)	Z=-6.446	**< 0.001**
LVEF status, n (%)				χ²=6.196	**0.013**
Normal	895 (89.77)	725 (90.97)	170 (85.00)		
Abnormal	102 (10.23)	72 (9.03)	30 (15.00)		
NT-proBNP, M (Q_1_;, Q_3_)	142.00 (60.84, 391.84)	133.96 (54.65, 353.00)	198.54 (99.00, 498.41)	Z=-4.018	**< 0.001**
HDL, M (Q_1_;, Q_3_)	1.16 (1.00, 1.35)	1.17 (1.01, 1.36)	1.12 (0.96, 1.30)	Z=-2.099	**0.036**
ALB, M (Q_1_;, Q_3_)	40.10 (37.50, 43.40)	40.20 (37.40, 43.40)	40.00 (37.77, 43.35)	Z=-0.010	0.992
EGFR, M (Q_1_;, Q_3_)	75.00 (64.00, 86.00)	74.00 (63.00, 85.00)	77.50 (65.00, 87.00)	Z=-1.688	0.091
Creatine, M (Q_1_;, Q_3_)	89.00 (77.00, 99.00)	89.00 (77.00, 99.00)	89.00 (77.00, 97.25)	Z=-0.159	0.874
TyG, Mean ± SD	8.56 ± 0.56	8.54 ± 0.55	8.68 ± 0.60	t=-3.187	**0.001**
TyG, n (%)				χ²=13.615	**0.001**
<8.67	608 (60.98)	501 (62.86)	107 (53.50)		
8.67–9.37	309 (30.99)	244 (30.61)	65 (32.50)		
>9.37	80 (8.02)	52 (6.52)	28 (14.00)		
Glucose, Mean ± SD	5.49 ± 1.53	5.44 ± 1.48	5.67 ± 1.69	t=-1.899	0.058
Triglyceride, M (Q_1_;, Q_3_)	1.19 (0.88, 1.67)	1.19 (0.87, 1.65)	1.21 (0.90, 1.84)	Z=-1.806	0.071
Medication use for arrhythmia upon discharge					
Class I antiarrhythmic drugs, n (%)				χ²=2.315	0.128
None	865 (86.76)	698 (87.58)	167 (83.50)		
Yes	132 (13.24)	99 (12.42)	33 (16.50)		
Class II antiarrhythmic drugs, n (%)				χ²=6.099	**0.014**
None	690 (69.21)	566 (71.02)	124 (62.00)		
Yes	307 (30.79)	231 (28.98)	76 (38.00)		
Class III antiarrhythmic drugs, n (%)				χ²=0.449	0.503
None	285 (28.59)	224 (28.11)	61 (30.50)		
Yes	712 (71.41)	573 (71.89)	139 (69.50)		
APPLE, Mean ± SD	1.29 ± 1.08	1.19 ± 1.05	1.68 ± 1.11	t=-5.897	**< 0.001**
CAAP-AF, Mean ± SD	3.46 ± 1.96	3.29 ± 1.88	4.16 ± 2.09	t=-5.420	**< 0.001**
Survival time, Mean ± SD	30.43 ± 17.75	34.03 ± 16.31	16.09 ± 15.98	t = 14.132	**< 0.001**

SD: standard deviation, M: Median, Q_1_;: 1st Quartile, Q_3_: 3rd Quartile

t: t-test, Z: Mann-Whitney test, χ²: Chi-square test, -: Fisher’s exact test

### Establishing trajectory models

The optimal trajectory category was determined by applying the principles of minimum BIC and maximum entropy. This classification process yielded three optimal categories, as presented in Table 2.

**Table 2:** LCMM model categories

Number of classes		Log likelihood	AIC	BIC	Entropy	% Class 1	% Class 2	% Class 3	% Class 4	% Class 5	
1		-1784.67	3583.35	3617.63	1	100					
2		-1763.29	3550.59	3609.36	0.222	75.96	24.04				
3	-1736.10	3506.21	3589.48	0.518	79.40	1.31	19.29				
4	-1729.69	3503.38	3611.13	0.490	63.23	21.62	1.52	13.64			
5		-1725.65	3505.29	3637.53	0.497	30.901	43.13	10.51	1.31	14.14	

The analysis of Table 2 reveals that the tripartite trajectory model exhibits the lowest BIC value (3506.21) and the highest entropy (0.518). Consequently, we select tripartite classification as the optimal number of trajectory classes for modeling.

The population distribution across various trajectories was computed in Table 3 based on the classification outcomes from Table 2. The findings revealed that Locus 1 comprised a population of 791 individuals, whereas Locus 2 and Locus 3 had populations of only 14 and 192 individuals, respectively.

**Table 3:** Distribution of populations in different trajectory groups

Class, n (%) *	Total (*n* = 997)	Non Recurence(*n* = 797)	Recurence(*n* = 200)	Statistic	P
Locus 1	791 (79.34)	654 (82.06)	137 (68.50)	-	**< 0.001**
Locus 2	14 (1.4)	9 (1.13)	5 (2.50)		
Locus 3	192 (19.26)	134 (16.81)	58 (29.00)		

**Fig. 2:**
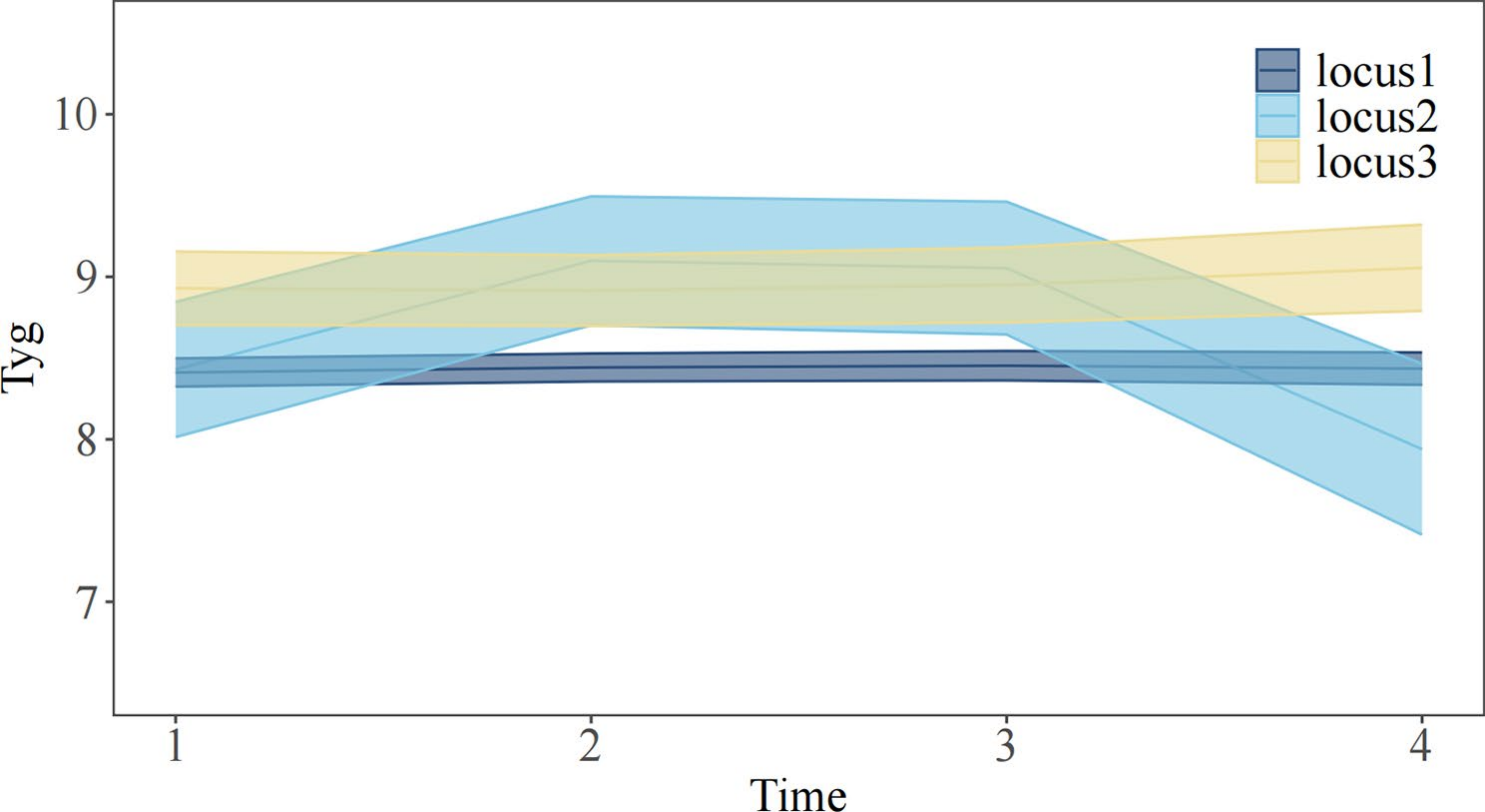
The trajectory trend chart

To visually enhance the presentation of the classified model trend, we have generated a trajectory trend graph. The trajectory trend can be categorized into three groups: Locus 1 shows minimal fluctuations over time; Locus 2 experiences notable periodic fluctuations with an initial rise followed by stability and subsequent decline, eventually falling below the starting point; Locus 3 demonstrates slightly increasing fluctuations compared to Locus 1, as depicted in Fig. 2.

The low-low group is represented by Locus 1, the low-high-low group is represented by Locus 2, and the high-high group is represented by Locus 3 in Fig. 2.

###  K‒M survival curves

**Fig. 3:**
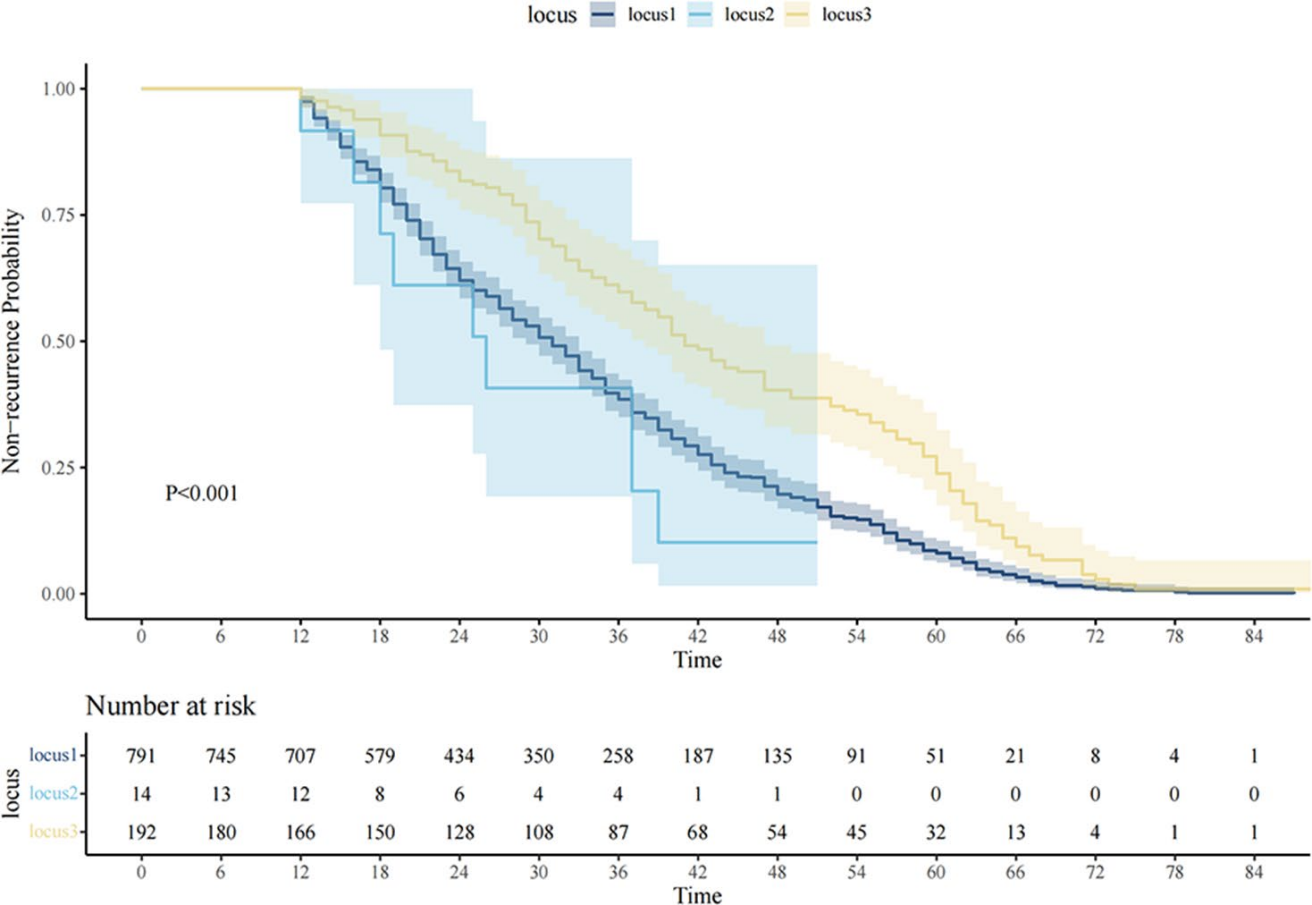
K‒M survival curve

The incidence rates of AF recurrence varied among the three tracks, as depicted in Fig. 3, and the differences between these groups were statistically significant. Regarding long-term outcomes, the high-high group exhibited a lower rate of AF recurrence.

### The univariable cox proportional hazards regression model

In the Univariable Cox regression model outlined in Table 4, it was observed that AF duration emerged as a significant risk factor for AF recurrence. Specifically, each incremental unit in onset duration was associated with a 1.18-fold increased risk (95% CI 1.063–1.310, *p* = 0.002). Left atrial diameter showed a notable association with an increased risk of AF recurrence, as each additional unit increase was linked to a 1.614-fold elevation in risk (95% CI 1.412–1.844, *p* < 0.001). NT-proBNP levels were identified as an independent predictor for recurrent AF, where each supplementary unit increase was connected to a 1.153-fold amplified risk (95% CI 1.058–1.257, *p* = 0.001). The classification of AF type also proved influential in AF recurrence, revealing that persistent AF bore a significantly higher risk compared to paroxysmal AF (HR = 2.180, 95% CI: 1.641–2.894, *p* < 0.001). The status of LVEF emerged as a significant prognosticator for AF recurrence; individuals with abnormal cardiac function exhibited an increased risk relative to those with normal cardiac function (HR = 1.758, 95% CI: 1.192–2.593, *p* = 0.004).

**Table 4:** Univariate Cox proportional hazards regression model

Exposure	Levels	HR (95%CI)	*P*
Age, years	-	1.078 (0.936–1.241)	0.299
Gender	Female	Reference	-
	Male	1.186 (0.882–1.595)	0.259
BMI, kg/m^2^	-	1.029 (0.894–1.184)	0.694
AF Duration, Months	-	1.180 (1.063–1.310)	**0.002**
Combined with other arrhythmias	No	Reference	-
	Yes	1.108 (0.775–1.585)	0.572
Hypertension	No	Reference	-
	Yes	1.094 (0.829–1.446)	0.525
SBP, mmHg	-	0.833 (0.619–1.120)	0.226
DBP, mmHg	-	1.073 (0.933–1.233)	0.323
CAD	No	Reference	-
	Yes	1.086 (0.728–1.620)	0.686
Diabetes	No	Reference	-
	Yes	0.992 (0.657–1.498)	0.969
Type of AF	Paroxysmal	Reference	-
	Persistent	2.180 (1.641–2.894)	**< 0.001**
CHA_2_DS_2−_VASc	-	1.091 (0.950–1.252)	0.217
HAS-BLED	-	1.097 (0.958–1.257)	0.181
Left atrial diameter, mm	-	1.614 (1.412–1.844)	**< 0.001**
LVEF status	Normal	Reference	-
	Abnormal	1.758 (1.192–2.593)	**0.004**
NT-proBNP, pg/mL	-	1.153 (1.058–1.257)	**0.001**
HDL, mmol/L	-	0.882 (0.751–1.036)	0.125
ALB, g/L	-	0.947 (0.823–1.090)	0.447
eGFR, mL/min/1.73 m^2^	-	1.044 (0.957–1.139)	0.333
Creatine, µmol/L	-	0.973 (0.849–1.115)	0.696

HR: hazard ratio, CI: confidence interval

### Baseline TyG and the Association between different trajectories and the risk of Atrial Fibrillation recurrence

After adjusting for age, sex, BMI, duration in months, type of AF, left atrial diameter, LVEF status, and NT-proBNP levels, the baseline Tyg level showed a positive correlation with AF recurrence (HR = 1.255, 95% CI: 1.087–1.448). Individuals with a TyG > 9.37 had a greater incidence of outcomes than did those with a TyG < 8.67 (HR = 2.056, 95% CI: 1.335–3.166). Additionally, individuals with Locus 3 had a greater incidence of outcomes than those with Locus 1 (HR = 1.580, 95% CI: 1.146–2.177), as indicated in Table 5.

**Table 5:** Baseline TyG and the association between different trajectories and the risk of AF recurrence

Exposure	Levels	Model 1			Model 2			Model 3	
		HR (95%CI)	P		HR (95%CI)	P		HR (95%CI)	P
Tyg	-	1.146 (1.002–1.311)	**0.047**		1.172 (1.019–1.348)	**0.026**		1.255 (1.087–1.448)	**0.002**
Tyg	<8.67	Reference	-		Reference	-		Reference	-
	8.67–9.37	1.133 (0.832–1.543)	0.428		1.156 (0.842–1.587)	0.370		1.277 (0.927–1.759)	0.135
	>9.37	1.726 (1.131–2.634)	**0.011**		1.792 (1.168–2.748)	0.008		2.056 (1.335–3.166)	**0.001**
Class	Locus 1	Reference	-		Reference	-		Reference	-
	Locus 2	2.433 (0.996–5.942)	0.051		2.197 (0.892–5.407)	0.087		2.131 (0.860–5.280)	0.102
	Locus 3	1.539 (1.128–2.099)	**0.006**		1.578 (1.150–2.165)	0.005		1.580 (1.146–2.177)	**0.005**

HR: Hazard Ratio, CI: Confidence Interval

Model 1 did not account for any covariates

Model 2 was adjusted for age, sex, and BMI

The variables adjusted in addition to Model 3 included AF duration, type of AF, Left atrial diameter, LVEF status, and NT-proBNP

### Subgroup analysis

The subgroup analyses were conducted based on age (cutoff: 65 years), sex, type of AF, presence or absence of diabetes, and LVEF (cutoff: 50%). The results are presented in Table 6.

**Table 6:** Subgroup Analysis

Subgroup	Exposure	Levels	HR (95%CI)	P
Age<= 65	Tyg	-	1.326 (1.088–1.617)	**0.005**
	Tyg	< 8.67	Reference	-
		8.67–9.37	1.314 (0.854–2.021)	0.214
		>9.37	2.641 (1.478–4.718)	**0.001**
	Class	Locus 1	Reference	-
		Locus 2	3.232 (0.781–13.385)	0.106
		Locus 3	1.867 (1.233–2.828)	**0.003**
Age > 65	Tyg	-	1.183 (0.955–1.465)	0.125
	Tyg	1	Reference	-
		2	1.242 (0.758–2.034)	0.390
		3	1.544 (0.786–3.035)	0.208
	Class	Locus 1	Reference	-
		Locus 2	2.086 (0.632–6.888)	0.228
		Locus 3	1.179 (0.699–1.988)	0.538
Female	Tyg	-	1.184 (0.920–1.523)	0.190
	Tyg	1	Reference	-
		2	1.260 (0.700–2.270)	0.441
		3	2.380 (1.187–4.768)	**0.015**
	Class	Locus 1	Reference	-
		Locus 2	0.000 (0.000-Inf)	0.997
		Locus 3	1.453 (0.843–2.505)	0.179
Male	Tyg	-	1.280 (1.076–1.523)	**0.005**
	Tyg	1	Reference	-
		2	1.276 (0.867–1.877)	0.217
		3	1.786 (1.009–3.158)	**0.046**
	Class	Locus 1	Reference	-
		Locus 2	2.154 (0.858–5.408)	0.102
		Locus 3	1.641 (1.096–2.458)	**0.016**
Paroxysmal AF	Tyg	-	1.320 (1.051–1.658)	**0.017**
	Tyg	1	Reference	-
		2	1.520 (0.912–2.533)	0.109
		3	2.396 (1.257–4.568)	**0.008**
	Class	Locus 1	Reference	-
		Locus 2	2.421 (0.573–10.235)	0.229
		Locus 3	1.838 (1.129–2.992)	**0.014**
Persistent AF	Tyg	-	1.147 (0.958–1.373)	0.136
	Tyg	1	Reference	-
		2	1.055 (0.694–1.604)	0.803
		3	1.550 (0.835–2.875)	0.165
	Class	Locus 1	Reference	-
		Locus 2	1.963 (0.606–6.354)	0.261
		Locus 3	1.269 (0.821–1.961)	0.283
Diabetes: No	Tyg	-	1.257 (1.079–1.464)	**0.003**
	Tyg	<8.67	Reference	-
		8.67–9.37	1.236 (0.875–1.747)	0.229
		>9.37	2.424 (1.492–3.938)	**< 0.001**
	Class	Locus 1	Reference	-
		Locus 2	2.609 (1.045–6.510)	**0.040**
		Locus 3	1.537 (1.077–2.195)	**0.018**
Diabetes: Yes	Tyg	-	1.508 (0.945–2.406)	0.085
	Tyg	<8.67	Reference	-
		8.67–9.37	1.686 (0.661–4.295)	0.274
		>9.37	2.105 (0.705–6.290)	0.183
	Class	Locus 1	Reference	-
		Locus 2	0.000 (0.000-Inf)	0.997
		Locus 3	2.844 (1.212–6.675)	**0.016**
LVEF normal	Tyg	-	1.330 (1.142–1.549)	**< 0.001**
	Tyg	<8.67	Reference	-
		8.67–9.37	1.406 (0.996–1.983)	0.053
		>9.37	2.235 (1.420–3.517)	**< 0.001**
	Class	Locus 1	Reference	-
		Locus 2	1.834 (0.576–5.836)	0.305
		Locus 3	1.565 (1.115–2.196)	**0.010**
LVEF abnormal	Tyg	-	0.624 (0.380–1.024)	0.062
	Tyg	<8.67	Reference	-
		8.67–9.37	0.409 (0.146–1.148)	0.090
		>9.37	0.683 (0.142–3.281)	0.634
	Class	Locus 1	Reference	-
		Locus 2	3.130 (0.633–15.492)	0.162
		Locus 3	1.623 (0.565–4.668)	0.369

HR: Hazard Ratio, CI: Confidence Interval

In the age ≤ 65 years, female sex, male sex, paroxysmal AF, diabetes status, diabetes status, and LVEF in the normal population, a greater incidence of adverse outcomes was observed in the Locus 3 group than in the Locus 1 group, indicating that Locus 3 is a significant risk factor.

In individuals aged ≤ 65 years, with paroxysmal AF, with diabetes, and with a normal LVEF, the presence of baseline TyG was a risk factor.

**Fig. 4:**
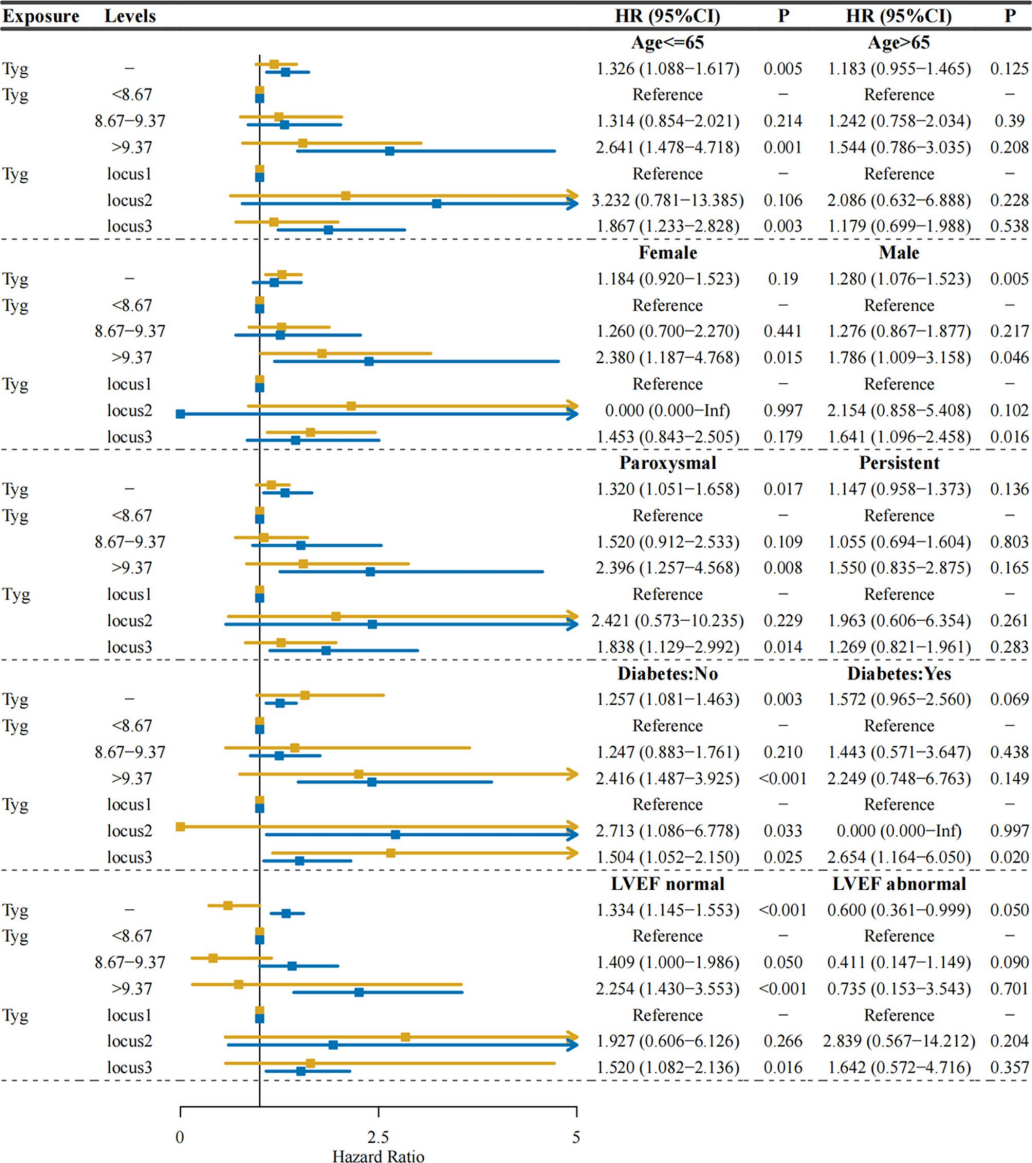
Subgroup forest graph

In the age ≤ 65, female, male, paroxysmal AF, diabetes: no, and LVEF normal population, the incidence of adverse outcomes was greater among individuals with a TyG > 9.37 than among those with a TyG < 8.67, indicating that the TyG level is a risk factor. Additional findings are presented in Table 6. The forest maps are provided specifically for subgroup analysis, as shown in Fig. 4.

In the forest plot, the blue line represents the categories analyzed by subgroups on the left, while the yellow line represents those analyzed by subgroups on the right. The baseline TyG was examined as the exposure, and the TyG was analyzed as an exposure factor using different Tyg Cutoff values. Additionally, the TyG was stratified by locus 1, locus 2, and locus 3 for further analysis.

### The predictive value of each indicator

**Fig. 5:**
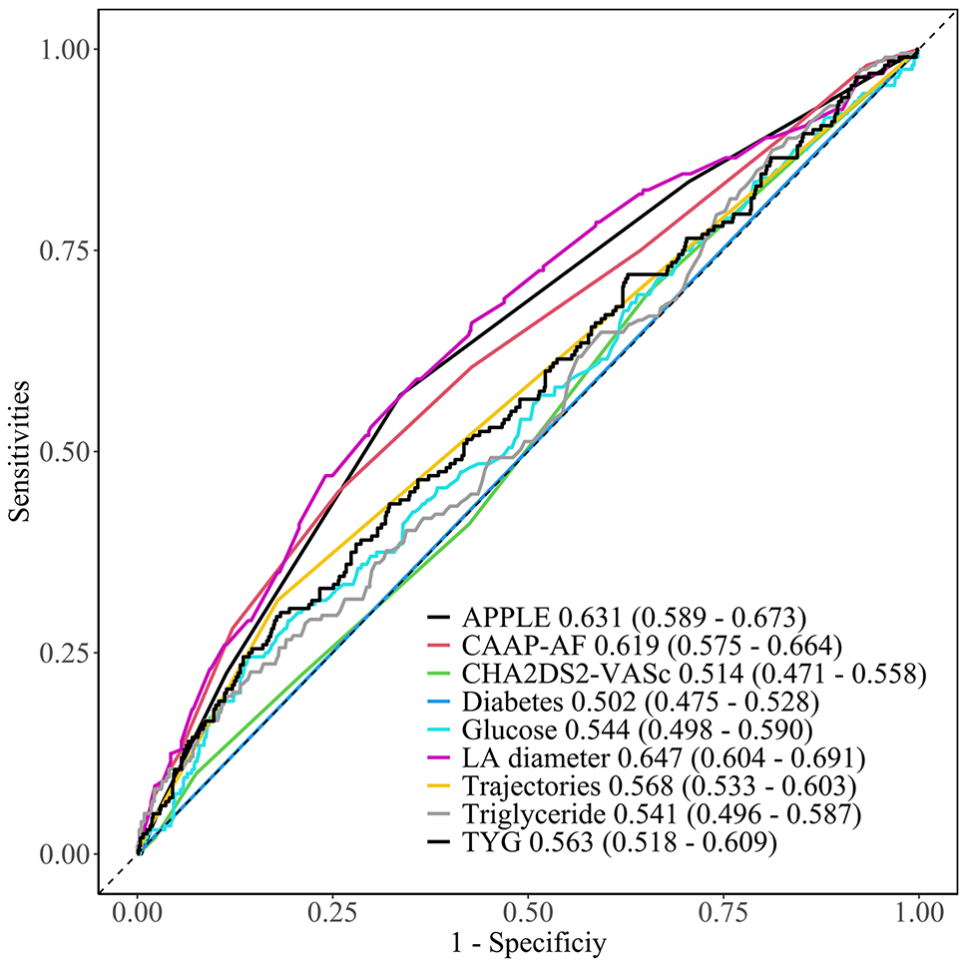
The predictive value of each indicator

Figure 5 shows that LA diameter has the highest predictive value for the outcome of AF recurrence, with an area under the curve (AUC) of 0.647 (95% CI 0.604–0.691). The remaining predictive indicators had the following values: the AUC of APPLE (0.631, 95% CI 0.589–0.673); the AUC of CAAP-AF (0.619, 95% CI 0.575–0.664); the AUC of CHA2DS2-VASc (0.514, 95% CI 0.471–0.558); the AUC of diabetes (0.502, 95% CI 0.475–0.528); the AUC of glucose (0.544, 95% CI 0.498–0.590); the AUC of trajectory (0.568, 95% CI 0.498–0.590); the AUC of triglycerides (0.541, 95% CI 0.496–0.587); and the AUC of TYG (0.563, 95% CI 0.518–0.609).

## Discussion

The retrospective study presented herein delineates three distinctive trajectory patterns observed by monitoring the TyG index during the blank period among a cohort comprising 997 patients who underwent AF RFCA. Notably, the TyG index was found to be an independent risk factor positively linked to AF recurrence, with locus 3 (high-high group) exhibiting an increased risk of recurrence relative to locus 1 (low-low group). Subsequent multivariable Cox regression analysis revealed that individuals with initial TyG levels exceeding 9.37 had an approximately twofold greater recurrence risk than those with initial TyG levels less than 8.67. Our study confirmed a significant correlation between the trajectory of the TyG index during the blanking period and AF recurrence outcomes in 3D AF patients.

The results of our study align with those reported by Azarboo A et al. [41]. Both the TyG index and Homeostasis Model Assessment of Insulin Resistance (HOMA-IR) index are commonly used in clinical practice to evaluate insulin resistance (IR) [42]. The HOMA-IR index is calculated as follows: HOMA-IR index = fasting insulin (U/dL) × fasting blood glucose (mmol/L)/22.5. However, gathering fasting insulin data in clinical settings can pose challenges. The hyperinsulinemic euglycemic clamp (HEC) is a commonly used method for measuring fasting insulin levels, but it is expensive, time-consuming, requires specialized equipment and personnel, and involves drawing nearly 300 ml of blood. Consequently, its use is limited to research applications with small sample sizes and not suitable for clinical practice or large-scale clinical studies. The TyG index, which relies on serum triglyceride testing rather than insulin testing, provides a simpler and more feasible method, particularly beneficial in resource-limited settings, and is favored by many clinicians.

Extensive clinical research has affirmed the reliability of the TyG index as a robust indicator of IR, showing significant associations with adverse cardiovascular events such as heart failure, in-stent restenosis, acute coronary syndrome, and arterial stiffness [43]. Similarly, IR also significantly influences the progression of AF. Recent studies by Azarboo A, Chen S, et al. have further confirmed the association between the TyG index and the occurrence of AF [41, 44]. The following factors contribute to the underlying mechanisms: (1) Cardiac Lipotoxicity: This condition is characterized by elevated levels of triglycerides and free fatty acids within cardiac tissue, resulting in excessive accumulation of intramyocellular triglycerides along with changes in lipid classes and their fatty acid profiles [45]. (2) Presence of advanced glycation end products (AGEs) in atrial tissues: A diminished capacity for tissue glucose metabolism results in the excessive accumulation of glucose, leading to the synthesis of AGEs. This process is likely to induce local inflammation and subsequently initiate irreversible structural and electrophysiological remodeling within the atria [46, 47].

It was discovered by Maria Z. et al., through animal experiments, that IR may induce GLUT protein expression on the surface of the atrial substrate, thereby increasing susceptibility to AF [29]. Ultimately, these changes result in structural modifications in the atrium that are irreversible and significantly impair ablative therapy’s ability to interrupt the vicious electrical cycle. Tang et al. demonstrated that elevated baseline TyG levels are associated with an increased risk of late AF recurrence [32].

BMI, Age, and Gender have been identified as strongly associated with AF recurrence [16, 22, 25, 48–50]. Additionally, factors like AF duration, AF type, left atrial diameter, and NT-proBNP are also acknowledged as contributors to AF recurrence [22, 51–53]. In our study, utilizing a univariate Cox proportional hazards model, we obtained results consistent with existing literature. Following adjustment for these risk factors, baseline TyG levels were identified as a risk factor for AF recurrence. Individuals with TyG levels exceeding 9.37 demonstrated a higher incidence of recurrent outcomes (HR = 2.056, 95% CI:1.335–3.166, *P* = 0.002). Subsequent ROC curve analysis explored the impact of TyG level and TyG trajectory on AF recurrence outcome. However, compared to traditional risk predictors for AF recurrence and established AF recurrence score prediction systems, the predictive value of TyG and its trajectory on AF outcome seems limited. Our findings suggest that AF recurrence post-ablation is a multifactorial outcome influenced by various factors, with TyG level or trajectory serving as just one of the risk factors. Nonetheless, our study underscores the potential benefit of aggressive intervention targeting insulin resistance in patients with stage 3D AF, which may contribute to an improved prognosis.

After AF ablation, patients experiencing recurrences during the “Blanking Period” are often considered benign, possibly linked to post-ablation atrial tissue edema and inflammation, typically not requiring immediate repeat ablation but emphasizing clinical monitoring [54, 55]. While previous studies have established a specific association between AF recurrences during the blanking period and delayed AF recurrences, there is a gap in research concerning longitudinal changes in relevant biochemical markers during this period regarding AF recurrence. Our recent investigation aimed to evaluate the significance of TyG trajectories during the blanking period in relation to 3D AF. The study findings indicate that elevated TyG trajectories (Locus 3: high-high group) are risk factors for an increase in AF recurrence after adjusting for confounding factors. Our Kaplan-Meier plot illustrates a higher risk of recurrence in the high-high group compared to the low-low group within the first year, consistent with prior studies [13, 53], as indicated by the “Number at Risk.” This suggests that reduced levels of insulin resistance may impede atrial electrical remodeling. However, over longer observation periods, Locus 3 exhibits a lower recurrence rate than Locus 1, which deviates from our current understanding. We propose that this inconsistency may potentially stem from skewed outcomes due to the limitations of our sample size.

In this subgroup analysis, an examination was conducted based on age using the Age criterion of 65 from the CHA2DS2-VASc stroke risk score in non-valvular atrial fibrillation (NVAF). It was observed that patients aged 65 or younger exhibited an increased risk of AF recurrence associated with higher TyG levels, regardless of trajectory and baseline TyG levels. This suggests that improving IR in younger patients may help delay atrial electrical remodeling and reduce AF recurrence. Concerning gender, elevated TyG levels consistently emerged as a significant risk factor for AF recurrence, irrespective of gender. Regarding AF subtype, increased TyG levels were associated with recurrence in paroxysmal AF but not in persistent AF. Prompt completion of ablation for paroxysmal AF in clinical practice is recommended to impede progression to persistent AF, interrupt the malignant electrical cycle, and prevent the development of a “low-voltage” AF substrate. The production of glycogen substrates on the atrial surface is induced by TyG, and controlling TyG levels after ablation aids in delaying atrial remodeling. Another subgroup analysis examined variances between diabetic and non-diabetic populations. In non-diabetic patients, an elevation in the TyG index was linked to AF recurrence, whereas such an association was not observed in diabetic individuals. Lee et al. identified a nonlinear relationship between insulin resistance and AF risk, whereby the incidence of AF rises when HOMA-IR levels are approximately 1-2.5 and then stabilizes [56]. It was also observed by Tang et al. that elevated TyG levels lead to an increased recurrence rate in non-diabetic patients [32]. Higher levels of insulin resistance in diabetic patients, such as increased HOMA-IR or glucose generation indices, may not be linked with AF risk [27, 48]. In terms of cardiac function, patients were categorized based on their left ventricular ejection fraction (LVEF) levels determined by echocardiography, using an LVEF cutoff point of 50%. They were classified into two groups: those with normal cardiac function and those with heart dysfunction. Controlling insulin resistance (IR) may have greater benefits in improving ablation outcomes during the 3D phase of AF with normal cardiac function. However, this study has limitations regarding heart failure as the impact of TyG on individuals with preserved ejection fraction was overlooked.

### Limitations

The study inevitably has some limitations. Firstly, despite obtaining data from two experienced AF centers, the sample size of AF cases remains relatively small, and a larger population cohort is needed to further validate the reliability of our findings and explore potential biological mechanisms more thoroughly. Secondly, due to patients undergoing AF RFCA, there was a limited proportion meeting the inclusion criteria for trajectory analysis. Thirdly, the lack of routine voltage monitoring during electrophysiologic procedures has prevented us from establishing a definitive correlation between TyG levels and low voltage. As a retrospective observational study, we could not establish a causal relationship between TyG and AF recurrence outcome. Additionally, this study did not investigate the impact of related antiarrhythmic drugs or secondary preventive drugs for cardiovascular diseases on AF recurrence during the Blanking period. Lastly, it is important to note that AF recurrence is influenced by multiple factors; thus, TyG provides us with novel diagnostic and treatment perspectives, while individualized treatment remains essential in clinical practice.

## Conclusion

The TyG trajectories of patients with stage 3D AF showed a strong association with AF recurrence outcomes. Monitoring TyG levels dynamically throughout follow-up can help identify high-risk patients prone to AF recurrence, enabling timely implementation of effective interventions.

### Electronic supplementary material

Below is the link to the electronic supplementary material.

Supplementary Material 1

## Data Availability

He derived data that were generated in the current study are available from the corresponding author upon reasonable request.

## Abbreviations

AF: Atrial fibrillation
AGEs: Advanced glycation end products
AUC: Area under curve
BIC: Bayesian information criterion
BMI: Body mass index
CAD: Coronary artery disease
CI: Confidence interval
Crea: Creatinine
DM: Diabetes mellitus
FBG: Fasting blood glucose
GLUT: glucose transporter
HEC: hyperinsulinemic euglycemic clamp
HF: Heart failure
HbA1c: Glycated hemoglobin
HR: Hazard ratio
HDL-C: High-density lipoprotein cholesterol
IR: Insulin resistance
LA: Left atrial
LDL-C: Low-density lipoprotein cholesterol
LVEF: Left ventricular ejection fraction
RFCA: Radiofrequency catheter ablation
ROC: Receiver operating characteristic
TC: Total cholesterol
TyG: Triglyceride and glucose
TG: Triglyceride
