# Supplementary material for: Association between triglyceride–glucose index trajectories and radiofrequency ablation outcomes in patients with stage 3D atrial fibrillation
Source: Cardiovasc Diabetol. 2024 Apr 5;23:121. doi: 10.1186/s12933-024-02219-w (PMC10998403; doi:10.1186/s12933-024-02219-w)
Supplement: Supplementary file 1 — Supplementary Material 1 [file 12933_2024_2219_MOESM1_ESM.docx]

| Variables | Missing ratio(N%) | Post-interpolation(n = 997) | Pre-interpolation (n = 997) | Statistics | P |
| --- | --- | --- | --- | --- | --- |
| BMI, Mean ± SD | 25 (2.50%) | 24.77 ± 3.12 | 24.76 ± 3.12 | t=0.061 | 0.951 |
| AF Duration M (Q₁, Q₃) | 3 (0.30%) | 12.00 (1.00, 36.00) | 12.00 (1.00, 36.00) | Z=-0.004 | 0.997 |
| SBP, M (Q₁, Q₃) | 6 (0.60%) | 128.00 (115.00, 140.00) | 128.00 (115.00, 140.00) | Z=-0.103 | 0.918 |
| DBP, M (Q₁, Q₃) | 6 (0.60%) | 79.00 (71.00, 86.00) | 79.00 (71.00, 87.00) | Z=-0.044 | 0.965 |
| CHA2DS2-VASC, M (Q₁, Q₃) | 1 (0.10%) | 2.00 (1.00, 3.00) | 2.00 (1.00, 3.00) | Z=-0.021 | 0.983 |
| HAS BLED, M (Q₁, Q₃) | 2 (0.20%) | 1.00 (0.00, 1.00) | 1.00 (0.00, 1.00) | Z=-0.014 | 0.988 |
| Left atrial diameter, M (Q₁, Q₃) | 39 (3.90%) | 39.00 (34.00, 43.00) | 39.00 (34.00, 44.00) | Z=-0.019 | 0.985 |
| LVEF, M (Q₁, Q₃) | 41 (4.10%) | 62.00 (57.00, 66.00) | 62.00 (57.00, 66.00) | Z=-0.164 | 0.870 |
| NT proBNP, M (Q₁, Q₃) | 23 (2.30%) | 142.00 (60.50, 390.47) | 143.00 (60.84, 392.00) | Z=-0.073 | 0.942 |
| HDL, M (Q₁, Q₃) | 5 (0.50%) | 1.16 (1.00, 1.35) | 1.16 (1.00, 1.35) | Z=-0.022 | 0.982 |
| ALB, M (Q₁, Q₃) | 2 (0.20%) | 40.10 (37.50, 43.40) | 40.10 (37.50, 43.40) | Z=-0.015 | 0.988 |
| Egfr, M (Q₁, Q₃) | 2 (0.20%) | 75.00 (64.00, 86.00) | 75.00 (64.00, 86.00) | Z=-0.054 | 0.957 |
| Cre, M (Q₁, Q₃) | 41 (4.10%) | 89.00 (77.00, 99.00) | 89.00 (77.00, 99.00) | Z=-0.076 | 0.939 |

**Suppelment**

1. **Random forest data interpolation**

**Tabel S1. Random forest data interpolation**

The variables with missing values in Table S1 include BMI, AF duration, SBP, DBP, CHA2DS2-VASC score, HAS-BLED score, left atrial diameter, LVEF, NT proBNP level, HDL level, ALB level, eGFR and creatinine. After applying the random forest method for interpolation, there was no statistically significant difference observed in these variables before and after (p > 0.05).
